# The Mechanism of Synchronous Precise Regulation of Two Shrimp White Spot Syndrome Virus Targets by a Viral MicroRNA

**DOI:** 10.3389/fimmu.2017.01546

**Published:** 2017-11-27

**Authors:** Yaodong He, Tiantian Ma, Xiaobo Zhang

**Affiliations:** ^1^Laboratory for Marine Biology and Biotechnology, Qingdao National Laboratory for Marine Science and Technology, College of Life Sciences, Zhejiang University, Hangzhou, China

**Keywords:** microRNA, target gene, cleavage, non-seed sequence, WSSV

## Abstract

MicroRNAs (miRNAs), important factors in animal innate immunity, suppress the expressions of their target genes by binding to target mRNA’s 3′ untranslated regions (3′UTRs). However, the mechanism of synchronous regulation of multiple targets by a single miRNA remains unclear. In this study, the interaction between a white spot syndrome virus (WSSV) miRNA (WSSV-miR-N32) and its two viral targets (*wsv459* and *wsv322*) was characterized in WSSV-infected shrimp. The outcomes indicated that WSSV-encoded miRNA (WSSV-miR-N32) significantly inhibited virus infection by simultaneously targeting *wsv459* and *wsv322*. The silencing of *wsv459* or *wsv322* by siRNA led to significant decrease of WSSV copies in shrimp, showing that the two viral genes were required for WSSV infection. WSSV-miR-N32 could mediate 5′–3′ exonucleolytic digestion of its target mRNAs, which stopped at the sites of target mRNA 3′UTRs close to the sequence complementary to the miRNA seed sequence. The complementary bases (to the target mRNA sequence) of a miRNA 9th–18th non-seed sequence were essential for the miRNA targeting. Therefore, our findings presented novel insights into the mechanism of miRNA-mediated suppression of target gene expressions, which would be helpful for understanding the roles of miRNAs in innate immunity of invertebrate.

## Introduction

MicroRNAs (miRNAs), a group of about 22 nucleotide non-coding small RNAs, participate in the regulation of the vast majority of life processes. In the innate immunity of animals and plants, miRNAs are very important factors by regulating the host and/or pathogen genes (1, 2). A mature miRNA is processed from a longer primary transcript by a series of ribonucleases, and then incorporated into RNA-induced silencing complex (RISC), in which it recognizes its target mRNAs by base pairing and guides RISC to cleave the mRNAs or inhibit the translation of mRNAs (1, 2). miRNA functions through the targeting of complementary nucleotide s in mRNA transcripts, usually in the 3′ untranslated region (3′UTR) of an mRNA. The identification and validation of mRNA–miRNA interaction is the basis for identifying the role of miRNA in the regulatory network of a broader range of biological processes. The number of potential target site is very large for the presence of a given miRNA (3–5). Therefore, the process of validation of potential miRNA targets in a laboratory is time consuming and expensive. Nowadays, the functions of many miRNAs have been identified (6–9). However, most of the existing reports focus on a single target gene of one miRNA (10–13). In fact, a single miRNA can target several genes by direct miRNA–mRNA interaction. As reported, multiple genes are the targets of miR-1/miR-206 in the progress of C2C12 myoblast differentiation (14). The melanoma cell invasion was suppressed through the downregulation of *c-Met* and *YB1* by MiR-137 (15). Multiple key components in human Hippo pathway, for example, *LATS2*, β*-TrCP, NDR2*, and *LZTS*, were target genes of miR-135b (16). In shrimp, white spot syndrome virus (WSSV) encodes viral miRNAs targeting one or two viral or host genes during virus infection (17, 18). Targets of viral miRNAs usually were identified as either viral regulatory proteins or proteins involved in the host immune system. For instance, viral T antigen of SV40 was a target of viral miR-S1. miR-S1 downregulated the viral T antigen to make sure cytotoxic T-cell would not attack the virus during late viral replication (19). Viral gene (HCMV IE1) is downregulated by miR-UL112-1 (20) was shown recently to express two miRNAs in latently infected neurons that are capable of downregulating ICP0 and ICP4 of HSV-1 were downregulated by its own miRNAs (21). Virus-induced apoptosis was inhibited by the downregulation of PUMA by miR-BART5 in EBV-infected cells (22). EBV miR-BHRF1–3 could target host chemokine, CXCL-11 (23). Another example, a few miRNAs of KSHV (miR-K5, miR-K9, and miR-K10) could knock down BCLAF1 (24). In human cancers, there is one-to-multiple relationship between a miRNA and its target genes (25). Although a single miRNA can target several genes, the interaction between a miRNA and its targets is evaluated using one miRNA–one target mRNA assay in all reports at present. Whether a single miRNA can simultaneously target different mRNAs in cells or/and *in vivo* has not been addressed yet. There is no report on the preference selection of target genes by a miRNA when the miRNA can target multiple genes.

To characterize the interaction between a miRNA and its multiple target mRNAs simultaneously, viruses may be the appropriate models. Viruses are the simplest organisms in the biological community (26, 27). A virus possesses a small genome and a short life cycle. The life cycle of a virus is finished in its host cells. Viruses, such as all living beings, have the ability to be genetic, mutated and evolutionary (28–30). As reported, some viral miRNAs can prevent host defense systems by targeting host genes (18, 19). More than half of the viral miRNAs are associated with virus infection. In this context, virus is one of the best models to investigate the miRNA–mRNA interaction.

To explore the interaction between a miRNA and its multiple genes *in vivo*, WSSV, a double-stranded DNA virus of shrimp, was employed in this study. Our previous studies have revealed that the viral miRNA WSSV-miR-N32 has two viral target genes (17, 18, 31). The results indicated that WSSV-miR-N32 could simultaneously target its target genes (*wsv459* and *wsv322*). The *wsv459* and *wsv322* genes, transcribed at the early stage of WSSV infection, played important roles in virus infection. The complementary bases (to the target mRNA) of a miRNA 9th–18th non-seed sequence were required for the miRNA targeting.

## Results

### Role of Viral miRNA WSSV-miR-N32 in Virus Infection

**Figure 1:**
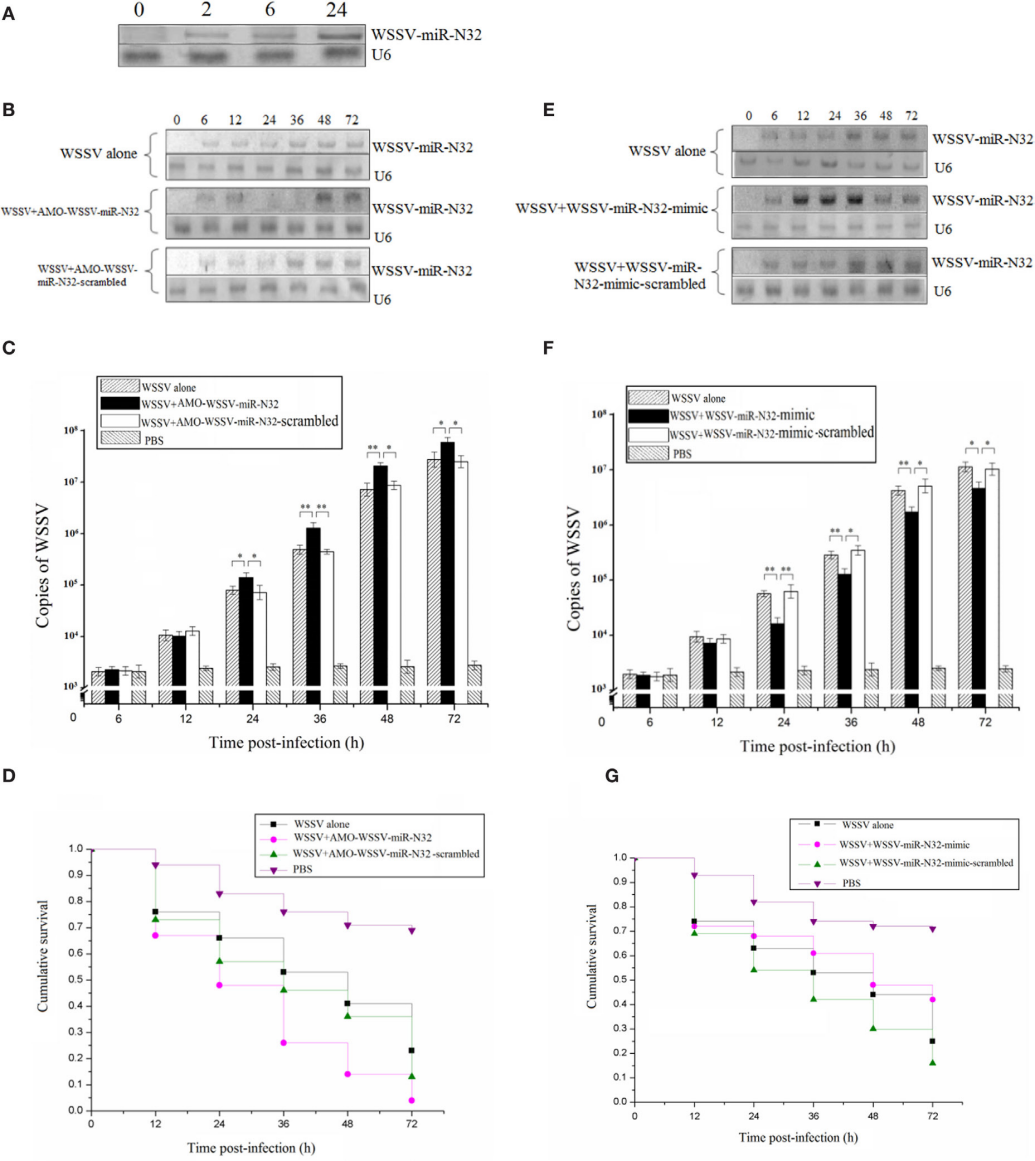
Role of white spot syndrome virus (WSSV)-miR-N32 in the virus infection. **(A)** The time-course detection of WSSV-miR-N32 in the WSSV-challenged shrimp. The shrimp were infected with WSSV. At different time postinfection, the expression of WSSV-miR-N32 in shrimp hemocytes was detected with Northern blots. The probes used were indicated at the right. U6 was used as a control. **(B)** The silencing of WSSV-miR-N32 in shrimp. Both WSSV and anti-miRNA oligonucleotide (AMO)-WSSV-miR-N32 or AMO-WSSV-miR-N32-scrambled were co-injected into shrimp. At different time points postinfection, the shrimp hemocytes were collected and subjected to Northern blot analysis. The probes were indicated at the right. U6 was used as a control. **(C)** The influence of WSSV-miR-N32 silencing on the WSSV copies in shrimp. Quantitative real-time polymerase chain reaction (PCR) was conducted to quantify the virus copies in shrimp treated with WSSV and AMO-WSSV-miR-N32 or AMO-WSSV-miR-N32-scrambled. For each treatment, three shrimp were randomly selected and the mixed RNAs of three shrimp were analyzed by quantitative real-time PCR. **(D)** The evaluation of shrimp cumulative survival. The treatments were shown on the top. The shrimp mortality was examined at different time after treatment. **(E)** The overexpression of WSSV-miR-N32 in shrimp. Shrimp were co-injected with WSSV and WSSV-miR-N32-mimic or WSSV-miR-N32-mimic-scrambled, followed by Northern blots to detect the WSSV-miR-N32 expression. **(F)** The quantification of WSSV copies in shrimp. The virus copies in shrimp were evaluated using quantitative real-time PCR. Three shrimp, selected at random for each treatment, were used for this analysis. The treatments were indicated on the top. **(G)** The shrimp survival analysis. After the overexpression of WSSV-miR-N32, the shrimp mortality was examined. In all panels, the significant differences between treatments were indicated with asterisks (*p < 0.05; **p < 0.01). The significance of difference between treatments was evaluated with Student’s *t*-test using the data of three independent assays for each treatment.

To investigate the role of the viral WSSV-miR-N32 in WSSV infection, the expression level of WSSV-miR-N32 in WSSV-challenged shrimp was investigated. WSSV-miR-N32 could be detected by northern blots as early as 2 h postinfection (Figure 1A), showing that this viral miRNA was transcribed at the very early phase of viral infection.

To evaluate the involvement of WSSV-miR-N32 in virus infection, the WSSV-miR-N32 expression was silenced or overexpressed in shrimp, followed by the examination of WSSV infection. The northern results showed that WSSV-miR-N32 was knocked down by AMO-WSSV-miR-N32 (Figure 1B). The WSSV-miR-N32 silencing caused major increases of WSSV copies (Figure 1C) and virus-infected shrimp mortality (Figure 1D). To overexpress WSSV-miR-N32 in shrimp, the WSSV-miR-N32-mimic and WSSV were co-injected into shrimp. Northern blots showed that WSSV-miR-N32 was significantly overexpressed in shrimp at 12–36 h postinfection compared with the controls (Figure 1E). The WSSV-miR-N32 overexpression resulted in significant decreases of WSSV copies and the survival of WSSV-infected shrimp (Figures 1F,G), indicating that WSSV-miR-N32 could inhibit the WSSV infection *in vivo*.

Collectively, these data revealed that WSSV-miR-N32 acted as a negative regulator during virus infection.

### Underlying Mechanism of WSSV-miR-N32 in Virus Infection

**Figure 2:**
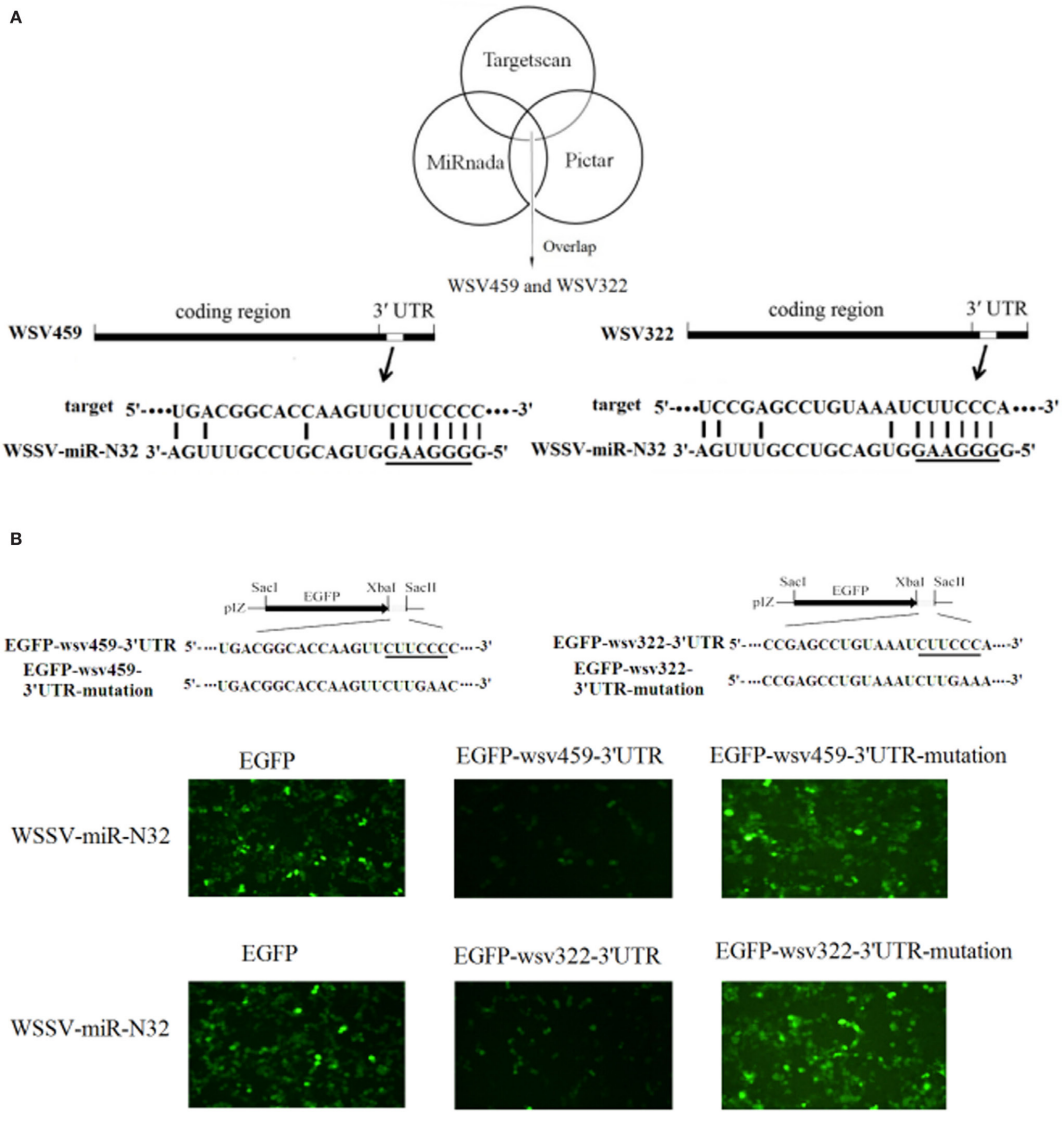

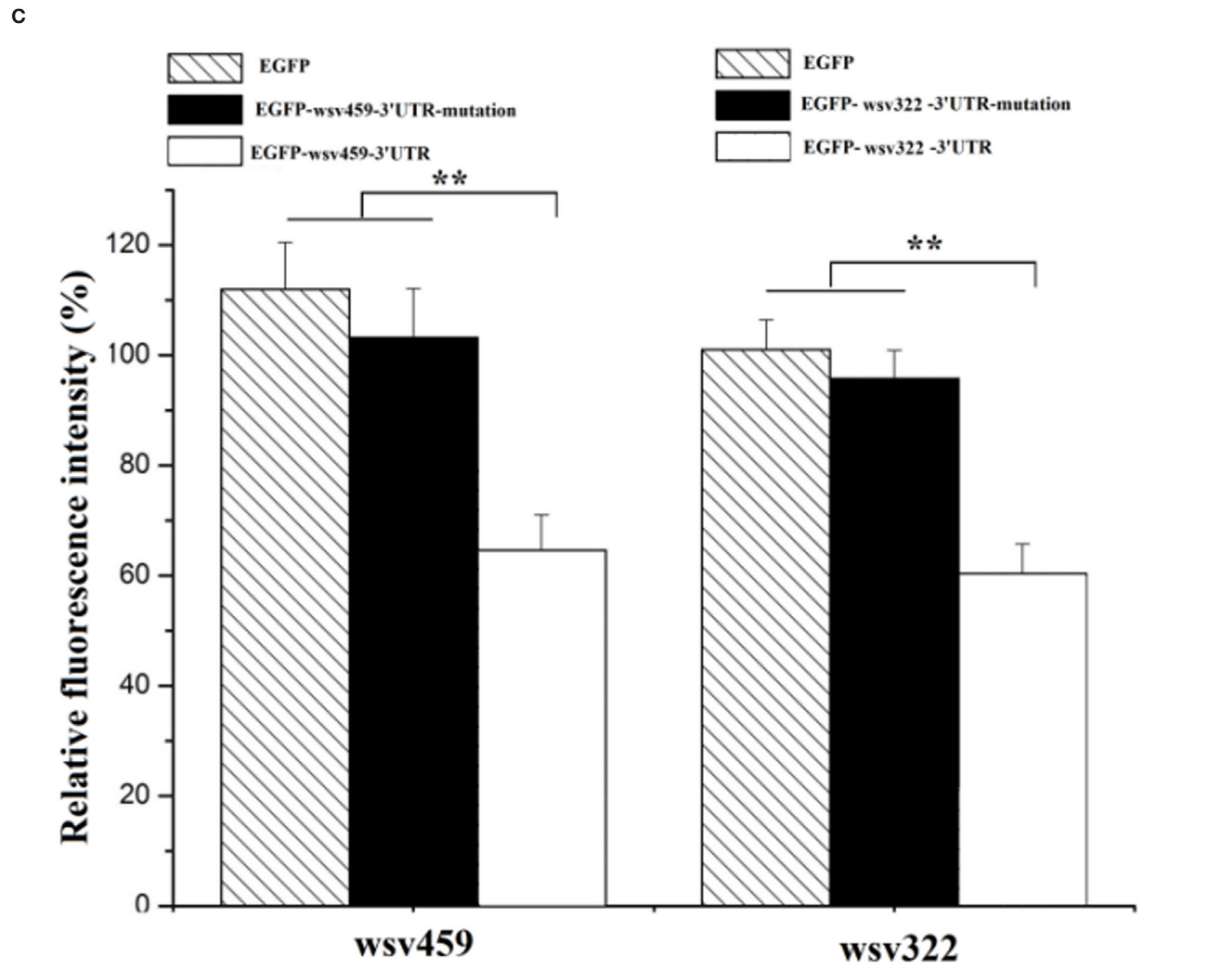

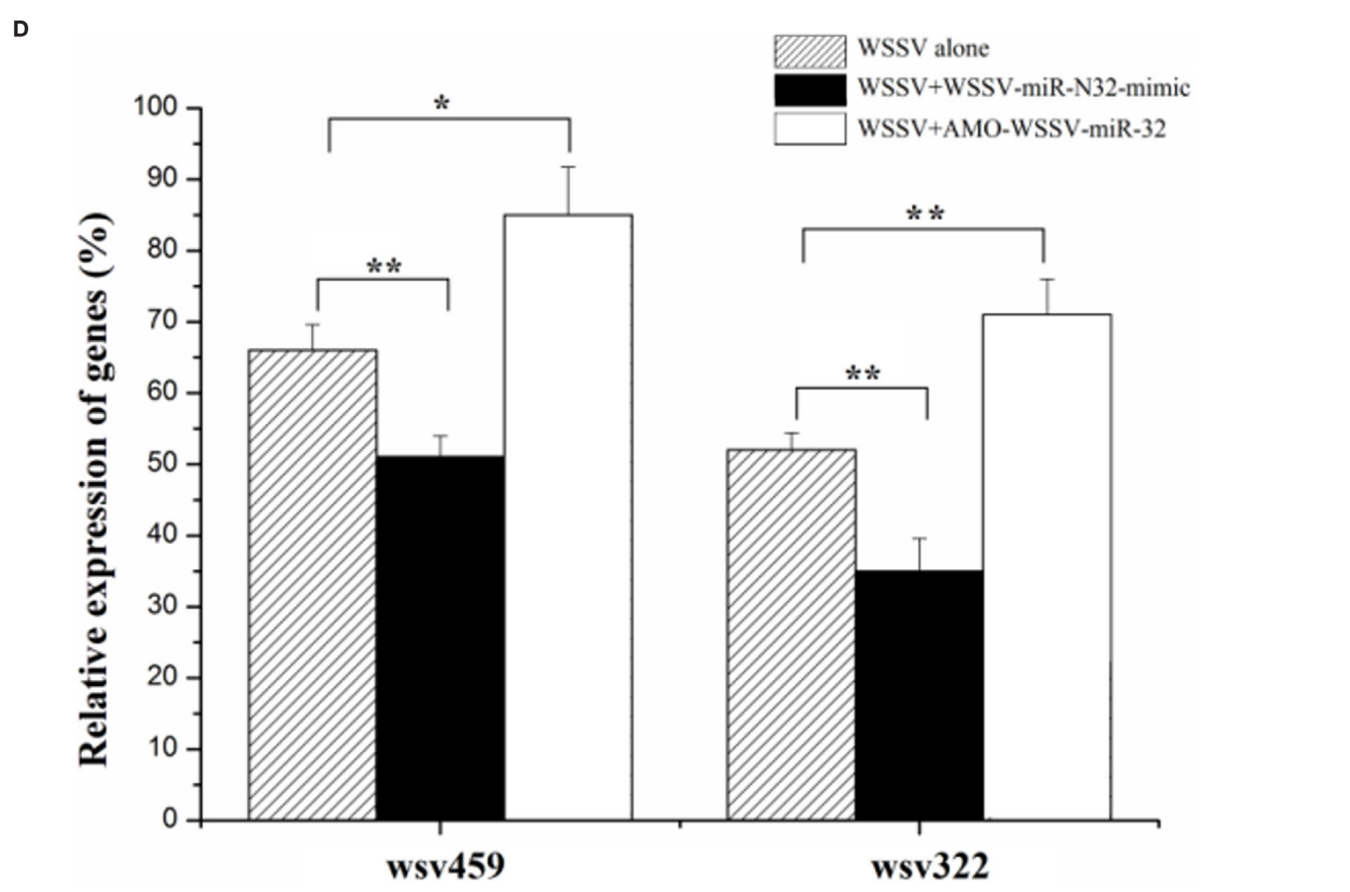
Mechanism of white spot syndrome virus (WSSV)-miR-N32 in virus infection. **(A)** Predicted target genes of WSSV-miR-N32. As predicted, the 3′ untranslated regions (3′UTRs) of the *wsv459* and *wsv322* genes were targeted by WSSV-miR-N32. The underline showed the seed sequence of WSSV-miR-N32. **(B)** The direct interactions between WSSV-miR-N32 and its target genes in insect cells. The insect High Five cells were co-transfected with WSSV-miR-N32 and enhanced green fluorescent protein (EGFP), EGFP-*wsv459*-3′UTR, EGFP-*wsv322*-3′UTR, EGFP-*wsv459*-3′UTR-mutation, or EGFP-*wsv322*-3′UTR-mutation. At 48 h after co-transfection, the fluorescence intensity of cells was examined. The sequences targeted by viral microRNA (miRNA) were underlined. **(C)** The effects of WSSV-miR-N32 on viral gene expressions. The relative fluorescence intensity of cells was determined. **(D)** The influence of WSSV-miR-N32 on viral gene expression *in vivo*. Shrimp were injected with WSSV and WSSV-miR-N32-mimic or anti-miRNA oligonucleotide (AMO)-WSSV-miR-N32. At 24 h after injection, the mRNA levels of *wsv459* and *wsv322* in shrimp hemolymph were examined with quantitative real-time polymerase chain reaction. Statistically significant differences between treatments were indicated by asterisks (*p < 0.05; **p < 0.01).

To explore the underlying mechanism of WSSV-miR-N32 in WSSV infection, the target genes of WSSV-miR-N32 were predicted. The prediction analysis revealed that two WSSV early genes (*wsv459* and *wsv322*) might be the targets of WSSV-miR-N32 (Figure 2A). To evaluate the interaction between WSSV-miR-N32 and its targets, WSSV-miR-N32 mimic and the 3′UTR of target gene were co-transfected into High Five cells (Figure 2B). The data revealed that the co-transfection of WSSV-miR-N32 and enhanced green fluorescent protein (EGFP)-*wsv459*-3′UTR or EGFP-*wsv322*-3′UTR significantly decreased the fluorescence intensity compared with the control, while the fluorescence intensity of cells co-transfected with WSSV-miR-N32 and EGFP-*wsv459*-3′UTR-mutation, or EGFP-*wsv322*-3′UTR-mutation did not change (Figures 2B,C). These findings indicated that WSSV-miR-N32 directly targeted *wsv459* and *wsv322* genes.

When WSSV-miR-N32 was overexpressed in WSSV-challenged shrimp, the expressions of viral *wsv459* and *wsv322* genes were significantly decreased *in vivo* compared with the control (Figure 2D). On the other hand, knocking down of WSSV-miR-N32 caused significant increases of *wsv459* and *wsv322* gene expressions in WSSV-infected shrimp (Figure 2D). These results indicated that WSSV-miR-N32 inhibited virus infection by directly targeting the viral genes *in vivo*.

### Functions of Viral *wsv459* and *wsv322* in Virus Infection

**Figure 3:**
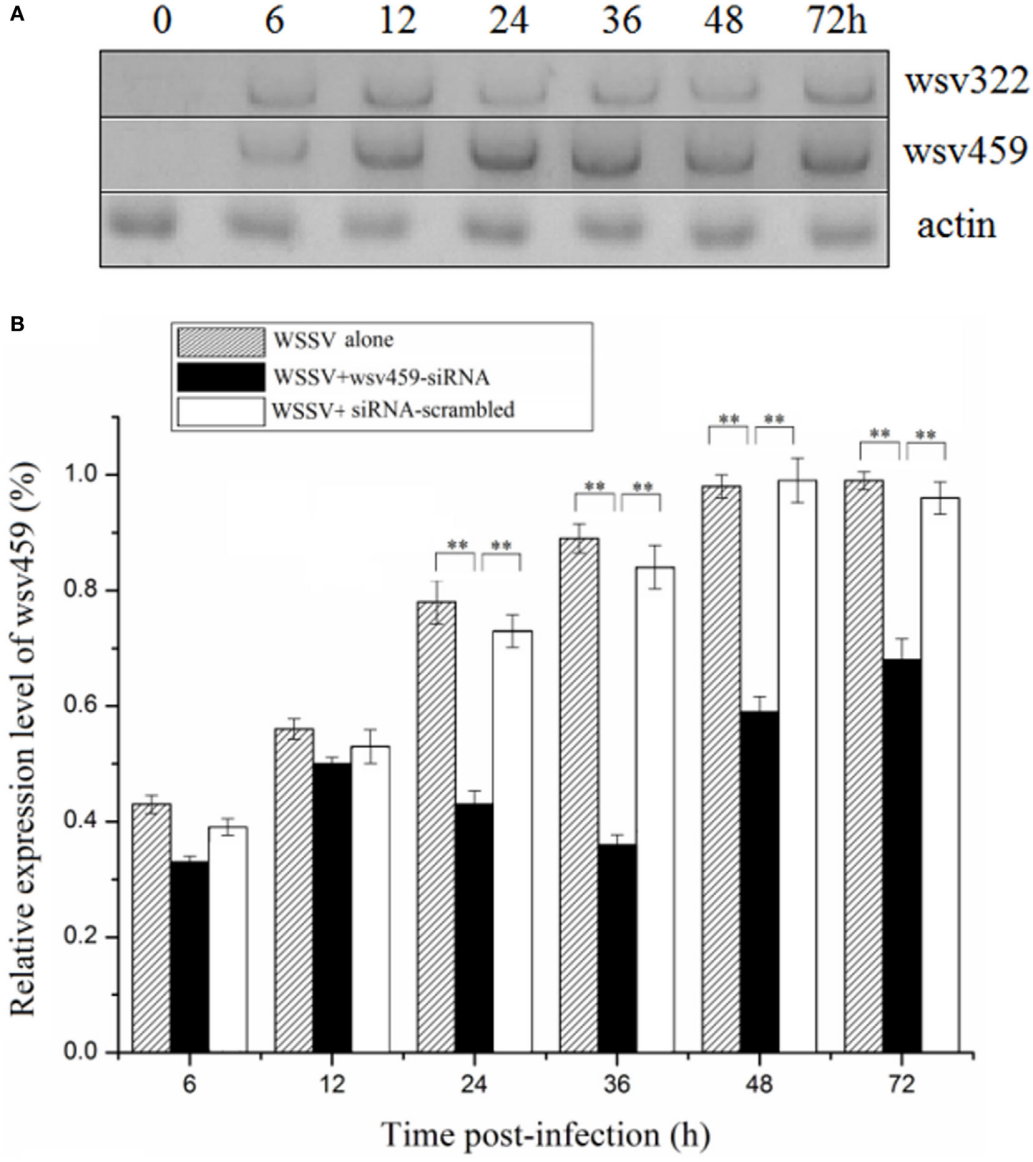

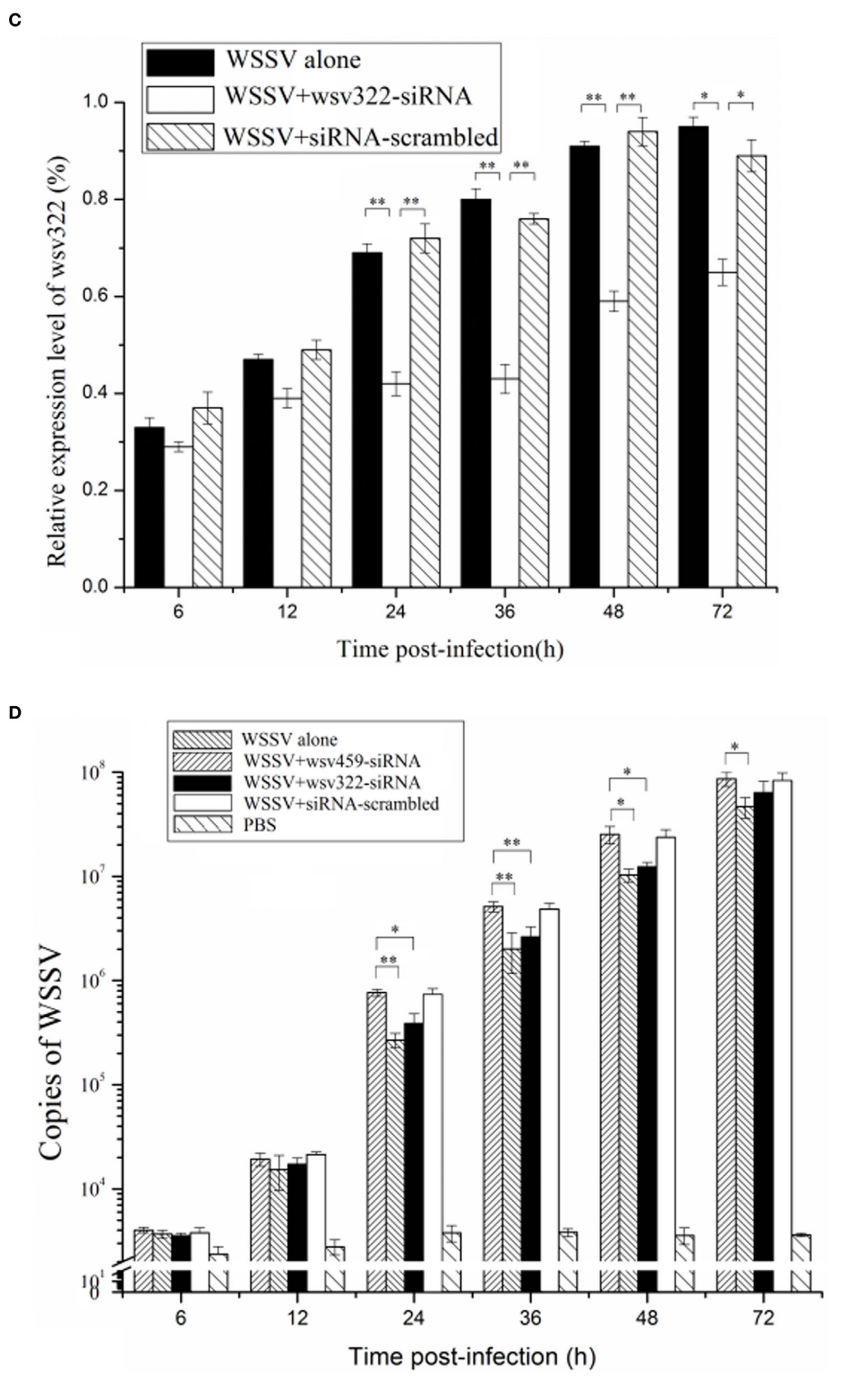

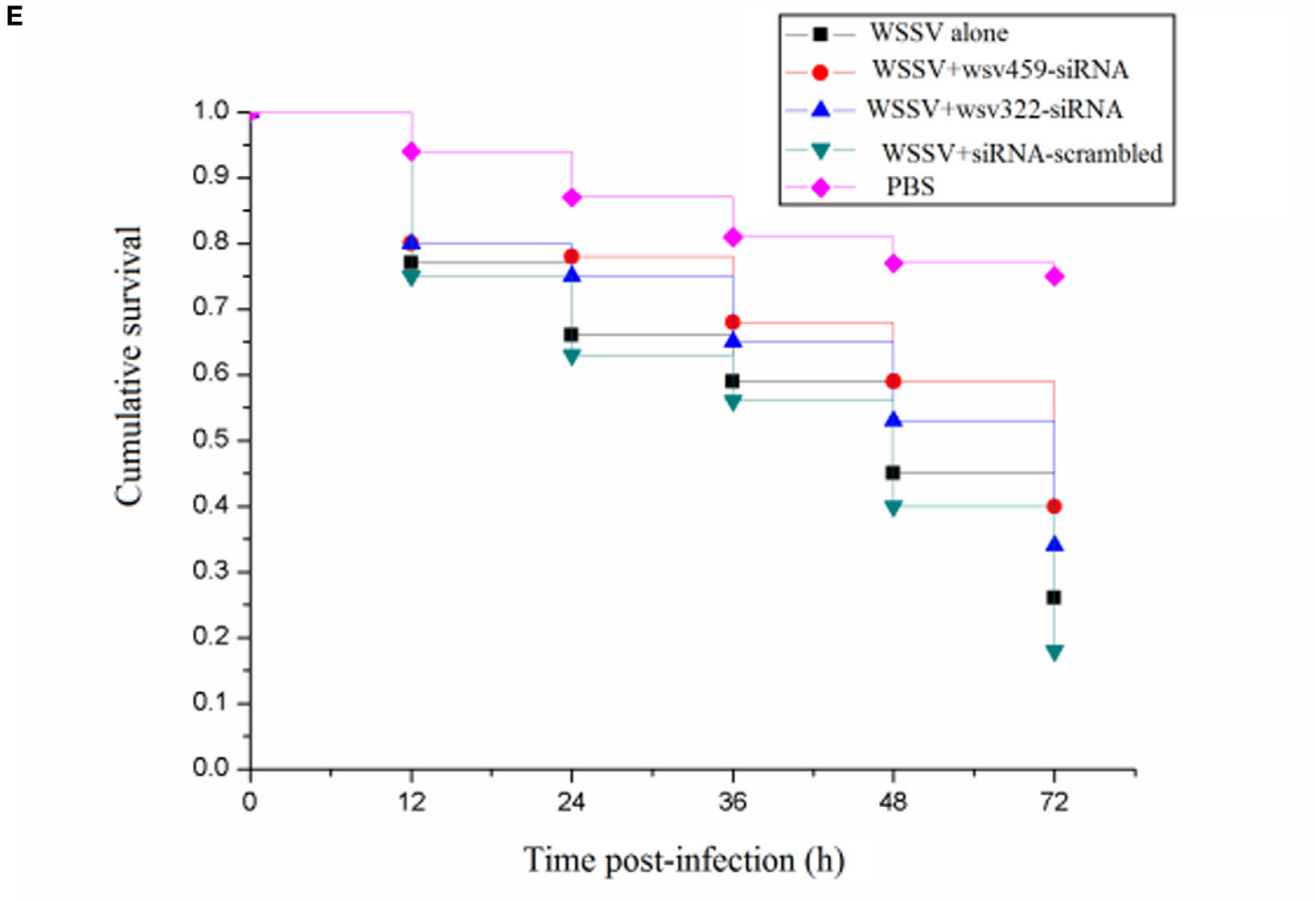
Functions of viral *wsv459* and *wsv322* in virus infection. **(A)** The expression levels of *wsv459* and *wsv322* in the white spot syndrome virus (WSSV)-infected shrimp. At different time postinfection, shrimp hemocytes were subjected to Northern blots. Actin was used as a control. **(B)** The silencing of *wsv459* gene expression in shrimp. WSSV and *wsv459-*specific siRNA (*wsv459-*siRNA) or siRNA-scrambled were co-injected into shrimp. WSSV alone was used as a control. At different time postinfection, the shrimp hemocytes were collected and subjected to quantitative real-time polymerase chain reaction (PCR) to detect the *wsv459* expression. **(C)** The knockdown of *wsv322* expression in shrimp. **(D)** The influence of *wsv459* or *wsv322* silencing on the WSSV infection in shrimp. The shrimp were co-injected with *wsv459-*siRNA and WSSV or *wsv322-*siRNA and WSSV. At various time postinfection, the WSSV copies of shrimp hemolymph were determined using quantitative real-time PCR. For each treatment, three shrimp were randomly selected for real-time PCR analysis. **(E)** The effects of *wsv459* or *wsv322* silencing on the survival of WSSV-infected shrimp. Shrimp mortality was examined at different time after treatment. In all panels, the significant differences between treatments were evaluated with Student’s *t*-test using the results of three independent experiments for each treatment and indicated with asterisks (*p < 0.05; **p < 0.01).

To reveal the roles of viral *wsv459* and *wsv322* genes in virus infection, the time-course expressions of the two genes in the WSSV-challenged shrimp were investigated. Northern blots indicated that the *wsv459* and *wsv322* mRNAs could be detected at 6 h postinfection (p.i.) onward (Figure 3A), showing that the two genes were both early genes.

To explore the functions of viral genes in virus infection, the expressions of *wsv459* and *wsv322* genes were silenced in the WSSV-infected shrimp *in vivo* by sequence-specific siRNAs, respectively. Quantitative real-time PCR data revealed that the mRNAs of *wsv459* and *wsv322* genes were depleted compared with the control groups (Figures 3B,C). When mRNAs of *wsv459* or *wsv322* was knocked down by siRNA, the copies of WSSV were significantly decreased and the WSSV-infected shrimp survivals were increased (Figures 3D,E), indicating that the two early genes of WSSV took great effects on virus infection.

These findings indicated that the viral *wsv459* and *wsv322* genes played positive roles in WSSV infection.

### Simultaneous Interaction between a miRNA and Its Two Targets

**Figure 4:**
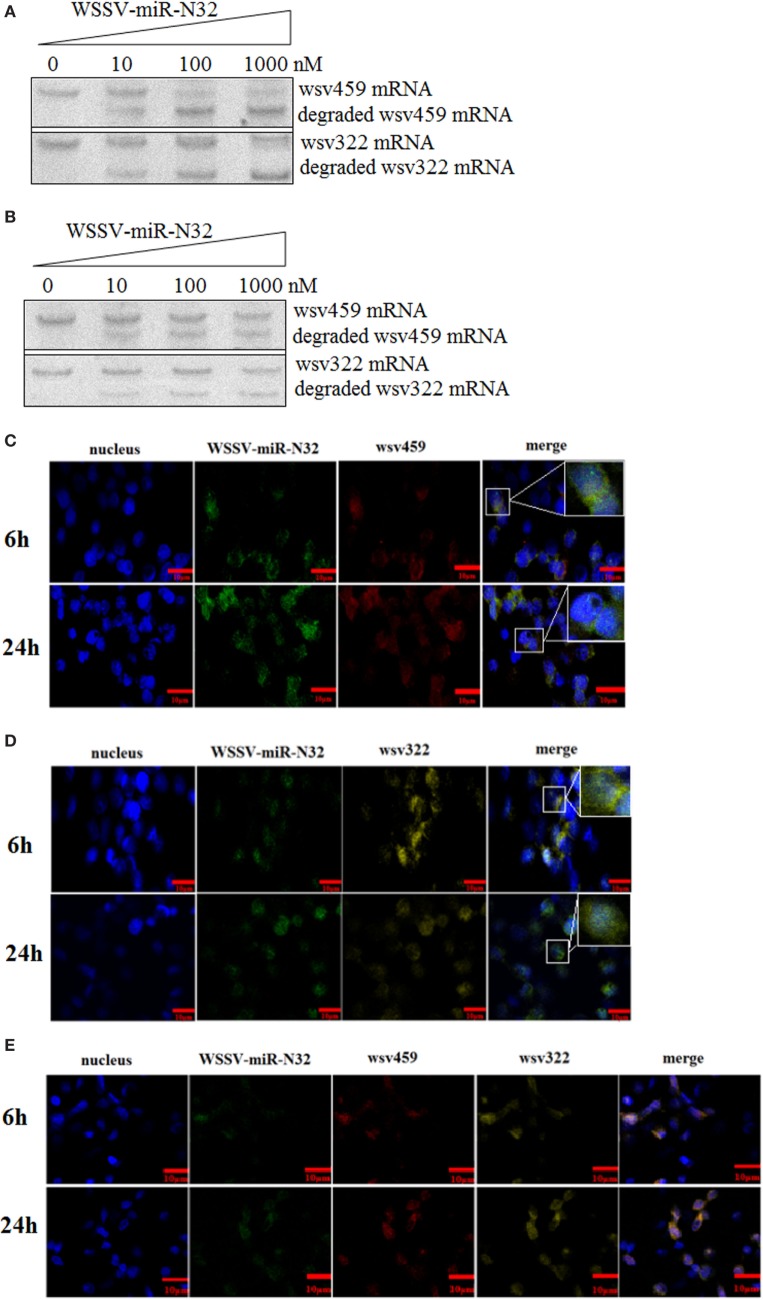
Simultaneous interaction between a microRNA (miRNA) and its two targets. **(A)** The miRNA-mediated degradation of target mRNA. White spot syndrome virus (WSSV)-miR-N32 at different concentrations was incubated with Ago1 complex and *wsv459*-3′UTR or *wsv322*-3′UTR for 2 h. Then the *wsv459* mRNA or *wsv322* mRNA was detected by Northern blot. **(B)** The simultaneous targeting of two mRNAs by WSSV-miR-N32. WSSV-miRN32 at various concentrations was incubated with the mixture of Ago1 complex, *wsv459*-3′UTR and *wsv322*-3′UTR for 2 h. Subsequently, the *wsv459* and *wsv322* mRNAs were detected by Northern blot. **(C)** The co-localization of WSSV-miR-32 and *wsv459* mRNA in the hemocytes of WSSV-infected shrimp. The shrimp were infected with WSSV. At 6 and 24 h postinfection, shrimp hemocytes were labeled using WSSV-miR-32-specific and *wsv459* mRNA-specific fluorescent probes. Scale bar, 10 µm. **(D)** The co-localization of WSSV-miR-32 and *wsv322* mRNA in the hemocytes of WSSV-infected shrimp. Scale bar, 10 µm. **(E)** The co-localization of WSSV-miR-32, *wsv459* mRNA, and *wsv322* mRNA in the hemocytes of WSSV-infected shrimp. Scale bar, 10 µm.

To explore the simultaneous interaction between WSSV-miR-N32 and its two targets (*wsv459* and *wsv322* genes), WSSV-miR-N32, the synthesized *wsv459* mRNA or *wsv322* mRNA, and the shrimp Ago1 complex were mixed, followed by the detection of *wsv459* mRNA or *wsv322* mRNA degradation. Northern blots demonstrated that WSSV-miR-N32 mediated the degradation of *wsv459* mRNA or *wsv322* mRNA in a miRNA-concentration-dependent manner (Figure 4A). When WSSV-miR-N32 was incubated with the mixture of *wsv459* mRNA, *wsv322* mRNA and Ago1 complex, the degraded *wsv459* mRNA and *wsv322* mRNA were detected (Figure 4B). The results indicated that WSSV-miR-N32 could simultaneously mediate the degradations of its two target mRNAs in the existence of its two targets.

To investigate the interactions between WSSV-miR-N32 and its two targets *wsv459* mRNA and *wsv322* mRNA *in vivo*, the localizations of WSSV-miR-N32, *wsv459* mRNA, and *wsv322* mRNA were conducted in WSSV-infected shrimp hemocytes. The results showed that WSSV-miR-N32 was co-localized with *wsv459* mRNA or *wsv322* mRNA in the hemocytes of WSSV-infected shrimp (Figures 4C,D). When WSSV-miR-N32, *wsv459* mRNA, and *wsv322* mRNA were simultaneously labeled with different probes, the fluorescent signals showed that the three molecules were co-localized (Figure 4E), indicating that WSSV-miR-N32 was interacted with *wsv459* mRNA and *wsv322* mRNA *in vivo*.

Stated thus, the above results suggested that a viral miRNA (WSSV-miR-N32) could simultaneously target its two targets (*wsv459* mRNA and *wsv322* mRNA) *in vivo*.

### Sites of a miRNA Non-Seed Sequence Required for miRNA Targeting

**Figure 5:**
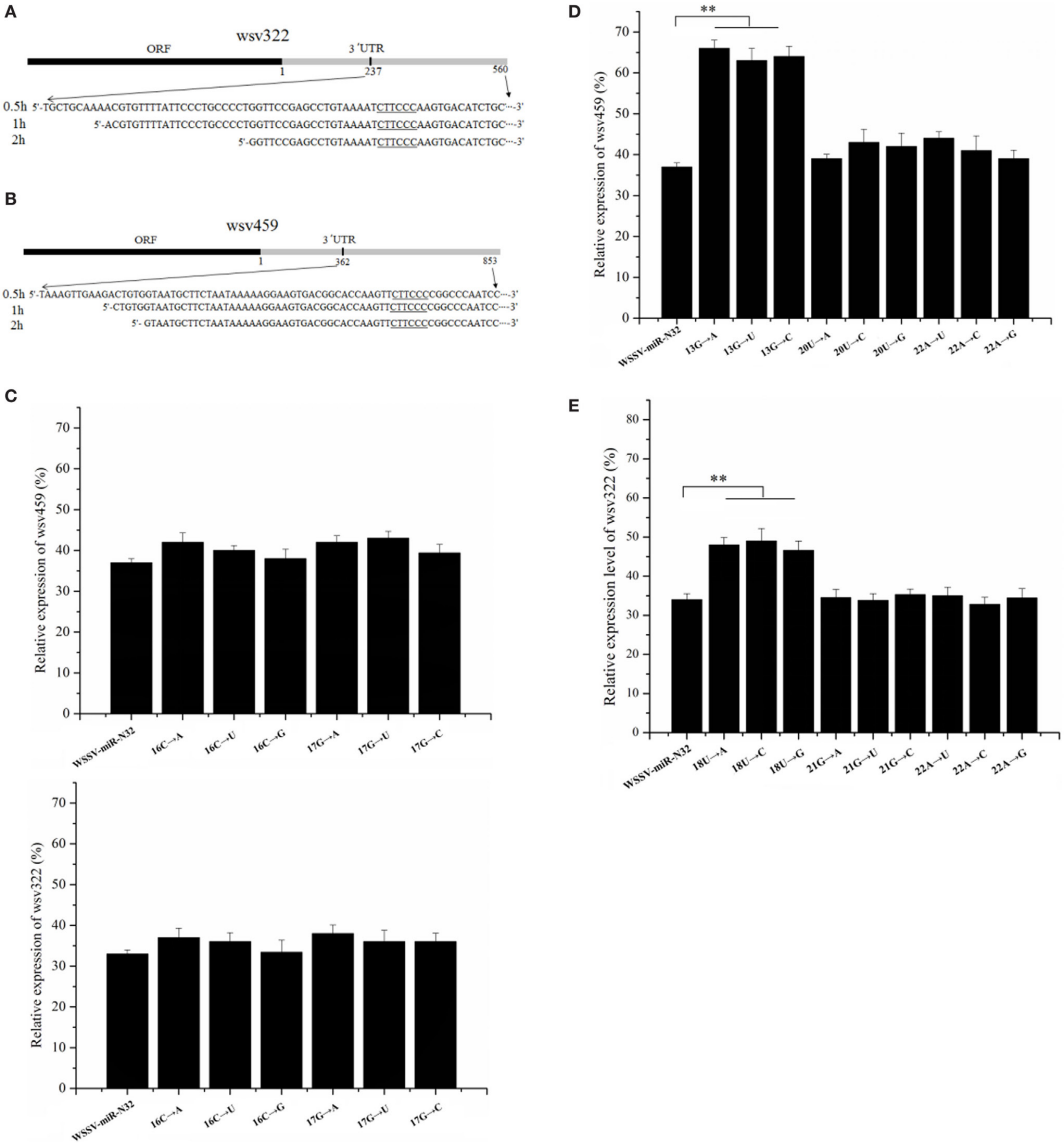
Sites of a microRNA (miRNA) required for miRNA targeting. **(A,B)** The miRNA-guided cleavage of target mRNA. White spot syndrome virus (WSSV)-miR-N32 was incubated with the Ago1 complex and 3′ untranslated region (3′UTR) of *wsv322* mRNA **(A)** or 3′UTR of *wsv459* mRNA **(B)** for different time as indicated on the left. Then, the cleaved fragment was sequenced. The sequence complementary to the seed sequence of WSSV-miR-N32 was underlined. **(C)** The influence of uncomplementary bases of WSSV-miR-N32 non-seed sequence on the expression of its target genes. The mutated or wild-type WSSV-miR-N32 was co-injected with WSSV into shrimp. The shrimp were cultured for 36 h. Then, the shrimp hemocytes were subjected to quantitative real-time polymerase chain reaction (PCR) to detect *wsv322* mRNA or *wsv459* mRNA. The arrows represented the mutated sites and bases of WSSV-miR-N32. **(D,E)** The effects of base mutation of WSSV-miR-N32 non-seed sequence on the expression of its target genes. WSSV and wild-type or mutated WSSV-miR-N32 were injected into shrimp. Thirty-six hours later, the *wsv322* mRNA level **(D)** or *wsv459* mRNA level **(E)** in shrimp hemocytes was examined with quantitative real-time PCR. The arrows indicated the mutated sites and bases of WSSV-miR-N32. In all panels, the statistically significant differences between treatments were shown with asterisks (**p < 0.01).

To further evaluate the sites of a miRNA required for the miRNA binding to its target mRNA, the cleaved fragments of *wsv459* mRNA and *wsv322* mRNA by WSSV-miR-N32-Ago 1 complex were sequenced. The results of time-course cleavage of 3′UTR showed that the *wsv322* mRNA 3′UTR was cleaved, generating the cleaved fragment containing the sequence complementary to the seed sequence of WSSV-miR-N32, while the 5′ end of *wsv322* mRNA 3′UTR was degraded (Figure 5A). The time-course cleavage of the *wsv459* mRNA 3′UTR yielded the similar results (Figure 5B). These data indicated that a miRNA could mediate 5′–3′ exonucleolytic digestion of its target mRNAs and this 5′–3′ exonucleolytic digestion stopped at the sites of target mRNA 3′UTRs close to the sequence complementary to the miRNA seed sequence.

It is well known that the seed sequence of a miRNA plays a decisive role in the interaction between miRNA and its target genes. As reported, except for the miRNA seed sequence, some bases (13th, 16th, 17th, 18th, 20th, 21st, and 22nd) of non-seed sequence may be required for the miRNA targeting of its target gene (6–8). To explore the sites of a miRNA non-seed sequence required for the miRNA targeting, 13th, 16th, 17th, 18th, 20th, 21st, or 22nd base of WSSV-miR-N32 was mutated, followed by injection of mutated WSSV-miR-N32 into WSSV-infected shrimp. The 13th, 20th, and 22nd bases of WSSV-miR-N32 were complementary to the *wsv459* mRNA sequence and the 18th, 21st, and 22nd bases of WSSV-miR-N32 were complementary to the *wsv322* mRNA sequence. The results showed that the 16th and 17th base mutations of WSSV-miR-N32 had no effect on the *wsv459* and *wsv322* gene expression (Figure 5C), indicating that the base of a miRNA non-seed sequence, which was not complementary to the target mRNA sequence, took no effect on the miRNA targeting. The quantitative real-time PCR data demonstrated that when the 13th G of WSSV-miR-N32 was mutated, compared with the control group, the *wsv459* mRNA level increased significantly (wild-type WSSV-miR-N32) (Figure 5D), indicating that the 13th G of WSSV-miR-N32 was required for the miRNA targeting. However, the 20th and 22nd base mutations of WSSV-miR-N32 had no effect on the *wsv459* gene expression (Figure 5D). The analysis of interaction between WSSV-miR-N32 and *wsv322* mRNA demonstrated that the 18th U of WSSV-miR-N32 was required for the miRNA targeting (Figure 5E). These data revealed that the complementary bases of a miRNA 9th–18th non-seed sequence, which were complementary to the target mRNA sequences, were required for the miRNA targeting.

## Discussion

Many studies have demonstrated that miRNAs play essential roles in virus–host interactions (17, 18, 32). They can regulate virus infection and host immunity by targeting viral or/and host genes. The seed sequence of a mature miRNA binding sites is complementary to mRNA seeds (3′UTR) *via* gene silencing by base pairing. As well known, a single miRNA can have multiple target genes. On the other hand, one gene could be targeted by several miRNAs. The complex regulatory network contributes to miRNAs’ fine control of the expressions of target genes (17, 33, 34). The gradual deepening of knowledge about the miRNA-regulated gene expression facilitates us to understand the complexity of the genome of higher eukaryotes and the complex gene expression regulation network. So far, however, nearly all the findings of miRNA–mRNA interactions are based on one (miRNA) to one (mRNA) reaction. The downregulation of miR-424 causes the upregulation of its target gene *Chk1* (35), whereas miR-99 regulates the DNA damage response through targeting the *SNF2H* gene (36). In prostate cancer, *CCND1* is a target of miR-193b (37). Our previous studies found that a viral miRNA WSSV-miR-N12 could target the virus *wsv399* gene, causing virus latency (31) and another virus-encoded miRNA WSSV-miR-N24 targeted the host *caspase 8* to repress the host apoptosis (18). WSSV-miR-22 could stimulate the virus infection by targeting the shrimp *STAT* gene in shrimp (38). Although there are some studies on multiple targets of one single miRNA, the miRNA–mRNA interactions are conducted using independent experiments of one (miRNA) to one (mRNA) reaction (11, 17, 39). However, the simultaneous regulation of multiple target genes’ expressions by a single miRNA has not been intensively investigated. In this study, the findings showed that a viral miRNA (WSSV-miR-N32) could synchronously regulate its two target genes’ expressions *in vivo*. The WSSV-miR-N32-guided Ago1 complex simultaneously cleaved mRNAs of two viral target genes (*wsv459* and *wsv322*), and the cleavage of *wsv459* mRNA and *wsv322* mRNA had no mutual interference. As the first line of immune responses, the innate immunity is fine regulated by miRNAs in animals (17, 18, 31, 38). During virus infection, viral miRNAs can target viral mRNAs. The targeting of viral genes by viral miRNAs leads to virus infection or virus latency, which can be an efficient strategy for virus to resist the innate immunity of host. Therefore, the mechanism of the interaction between a single viral miRNA and its multiple target mRNAs, which was revealed in this study, contributed novel insights into the innate immunity of animals against virus infection.

At present, there is no unified conclusion about the effects of miRNAs in the RISC on their target mRNAs. Some reports show that a miRNA completely complementary or almost completely complementary to its target mRNA can guide the mRNA degradation (32, 40, 41). However, some studies indicate that the cleavage of target mRNA takes place as long as the seed sequence of a miRNA is complementary to its target mRNA (42, 43). It is well known that RISC is a cytoplasmic effector of the miRNA pathway, in which a single stranded miRNA directs it to its target mRNA (44). In the RISC, Ago protein is the core component, which cleaves mRNAs. The findings revealed in this study and previous studies (42, 43) show that the 3′ fragment of RISC-cleaved mRNA can be detected, indicating that the 5′ fragment of mRNA is degraded. During the cleavage process of mRNA in the RISC, Ago acts as a 5′ phosphomonoester-producing RNA endonuclease (42, 43). In this study, the results indicated that a miRNA could direct the 5′–3′ exonucleolytic cleavage of its target mRNA, which stopped at the sites of target mRNA 3′UTR close to the sequence complementary to the miRNA seed sequence, and the complementary bases (to the target mRNA sequence) of a miRNA 9th–18th non-seed sequence were required for the miRNA targeting. In this context, this investigation provided novel insights into the mechanism of miRNA–mRNA interactions in miRNA-induced silencing complex.

## Materials and Methods

### Shrimp (*Marsupenaeus japonicus*) Culture and Virus Infection

Each shrimp (*M. japonicus*) was cultured in 80-L aquariums at 20–25°C. For each treatment, 20 shrimp were used. To ensure the absence of WSSV before experimental infection, three shrimp, selected at random, were subjected to the detection of WSSV using PCR with WSSV-PCR-primers (forward 5′-TATTGTCTCTCCTGACGTAC-3′ and reserve 5′-CACATTCTTCACGAGTCTAC-3′). The total DNA was extracted from shrimp using SQ tissue DNA kit (Omega Bio-Tek, USA) according to the manufacturer’s manual (17).

100 µL of WSSV (10^5^ copies/mL) inoculum/shrimp was injected into each virus-free shrimp (17). Shrimp hemocytes were gathered for later use at different time points postinfection (17, 18).

All the animal experiments were conducted according to the ethical requirements and were approved by the Experimental Animal Centre of Zhejiang University, China [Experimental animal license: SYXK (Zhejiang) 2012-0178].

### Northern Blot Analysis

The miRNA expression level was determined with Northern blot. Total RNAs were extracted from shrimp hemolymphs by mirVanaTM miRNA isolation kit (AM1561) (Ambion, USA) (17, 18).

RNAs were separated by a 15% polyacrylamide gel (denatured with 8 M urea). After that, the RNAs were transferred to a nylon membrane (GE Amersham, USA) (17, 18, 31, 38).

Then, the membrane was hybridized with a DIG-labeled probe (WSSV-miR-N32, 5′-TCAAACGGACGTCACCTTCCCC-3′; U6, 5′-GGGCCATGCTAATCTTCTCTGTATCGTT-3′).

The expression of *wsv459* or *wsv322* gene was detected using Northern blot. The extraction and hybridization of RNA were conducted as described earlier. The DIG-labeled sequence-specific probes (*wsv459* probe, 5′-AGACGCCACCAATGGCGAA-3′; *wsv322* probe, 5′-AGAAGTGGATGATGACGTTGA-3′; actin probe, 5′-C TCGCTCGGCGGTGGTCGTGAAGG-3′) were used.

### Silencing or Overexpression of WSSV-miR-N32 in Shrimp

To knock down the expression of WSSV-miR-N32, the sequence-specific anti-miRNA oligonucleotide (AMO) with a phosphorothioate backbone and a 12th nucleotide-2′-*O*-methyl modification was used. The AMO-WSSV-miR-N32 (5′-TCA AACGGACGTCACCTTCCCC-3′) and the control AMO-WSSV-miR-N32-scrambled (5′-TCAAACGGACGTCACCCCTTCC-3′) were synthesized (Sangon Biotech, Shanghai, China). WSSV (10^5^ copies/mL) and AMO (10 nM) were co-injected into shrimp (100 μL/shrimp). At 12 h after the co-injection, the AMO (10 nM) was injected into the same shrimp. At different time after the first injection (0, 2, 4, 6, 12, 24, 36, 48, and 72 h), the hemolymph of three shrimp, selected at random, was collected. Then, Northern blot was carried out, and WSSV copies were detected. We monitored the mortality of shrimp every day. All experiments were repeated three times.

To overexpress WSSV-miR-N32, the synthesized WSSV-miR-N32-mimic was injected into shrimp. According to the manufacturer’s manual, WSSV-miR-N32-mimic (5′-GGGGAAGGUGACGUCCGUUUGA-3′) was synthesized using T7 Kit siRNA Synthesis (TaKaRa, Japan). WSSV-miR-N32-mimic-scrambled (5′-GGAAGGGGUGACGUCCGUUUGA-3′) was used as a control. The synthesized miRNAs would be dissolved in Tris buffer (50 mM Tris·HCl (pH 7.5), 100 mM NaCl), then quantified by NanoDrop 2000. WSSV (10^5^ copies/mL) and a miRNA mimic (30 nM) were co-injected into shrimp. 12 h later, the miRNA (30 nM) was injected into the same shrimp. PBS and WSSV only (10^5^ copies/mL) were used as controls. At various times after first injection, shrimp hemolymph was collected for later use.

### Detection of WSSV Copies Using Quantitative (Q) Real-time PCR

Shrimp hemocytes of various treatments were collected at different times postinfection. According to the manufacturer’s manual, viral DNA was extracted using a Tissue DNA Kit (Omega Bio-Tek, USA). Then, the copies of WSSV were evaluated using quantitative PCR (qRT-PCR) with specific primers of WSSV (forward 5′-TTGGTTTCAGCCCGAGATT-3′ and reverse 5′-CCTTGGTCAGCCCCTTGA-3′) and specific TaqMan probe of WSSV (5′-FAM-TGCTGCCGTCTCCAA-TAMRA-3′). The mixture (25 µL) of PCR reaction consisted of 200 ng viral genomic DNA, 100 nM of TaqMan probe, 200 nM of each primer, and 12.5 µL 2× PCR Mix buffer. The PCR reaction was conducted at 95°C (1 min), and followed by 40 cycles of 95°C (30 s), 52°C (30 s), and then 72°C (30 s).

### Mortality of Shrimp

The shrimp were cultured with different treatments (20 shrimp per treatment). The cumulative shrimp mortality was examined at different time postinfection (12, 24, 36, 48, and 72 h).

### Prediction of Target Genes of miRNA

To get the target sequences of a miRNA, the genome of WSSV (GenBank accession no: AF332093) was used. The target genes of a miRNA were predicted using three independent bioinformatic softwares including pictar,^1^ miRanda,^2^ and TargetScan 5.1.^3^ The overlapped genes of three algorithms might be the targets of a miNRA.

### Fluorescence Plasmid Construction

PIZ/EGFP V5-His plasmid (Invitrogen, USA) that contains an EGFP gene was used for the plasmid construction. Primers used as follows: EGFP-*wsv459*-3′UTR (primers forward 5′-AACTCTAGAATCTAATTACAGAGTATTCTA-3′ and reverse 5′-AACCGCCGACATGATCCAACTGCATAGTCACCCGTAT-3′) and EGFP-*wsv322*-3′UTR (primers forward 5′-AACTCTAGAAATTCTGGCTACCAGAACCCA-3′ and reverse 5′-AACCGCCGCGTTGCAAGGAAACAGTGCTCAATACCT-3′). Each 3′UTR was linked into downstream of EGFP of the PIZ/EGFP plasmid. SacII and XbaI restriction sites were used. Dpn I-mediated site-directed mutagenesis (New England BioLabs, USA) were used to obtain the mutations. Generally, two incomplete complementary primers were used to mutate the 3′UTR sequence by PCR. The incomplete complementary primers contained two mismatched nucleotides. Dpn I was added into the PCR product. Then the mixture was incubated at 37°C (1 h). After that, DNA was purified and transformed into competent cells DH5α. All the sequences of above vectors were confirmed by DNA sequencing.

### High Five Cell Culture, Transfection, and Fluorescence Assays

At temperature of 27°C, *Trichoplusia ni* cell line High Five (Invitrogen, USA) was cultured in Express Five medium (Invitrogen, USA) with 1.8 mM/mL l-glutamine (Invitrogen, USA) were cultured. The cells were co-transfected with a synthesized miRNA mimic (300 nM) and pIZ/EGFP consisting of 3′UTR of *wsv459* or *wsv322* (6 µg/mL) at about 70% confluence. The miRNA mimic was synthesized by T7 Kit siRNA Synthesis (TaKaRa, Japan). All experiments of transfection were accomplished with Cellfectin (Invitrogen) according to Cellfectin’s manual in triplicate. 12 h for after culture, cells were planked with cell density of 2.0 × 10^4^ cells/well to 96-well plates. The fluorescence density of High Five was detected using a Flex Station II microplate reader (Molecular Devices, USA) with condition of Ex (490 nm)/Em (510 nm) at 48 h post-transfection. The fluorescence value was revised by subtracting the spontaneous fluorescence of non-EGFP cells.

### Quantification of mRNA with Real-time PCR

Total RNAs were extracted from shrimp hemocytes using a commercial RNA kit AM1640 (Ambion, USA). The cDNA was reverse transcripted using PrimeScript RT Reagent Kit (Perfect Real Time) (TaKaRa, Japan). Real-time PCR was performed with DRR390A Probe qPCR (TaKaRa, Japan) using sequence-specific primers (*wsv459*, 5′-CAAGGCTCCTCTCTTAGCATC-3′ and 5′-GTATTGATCCCAGCGCAGA-3′; *wsv322*, 5′-TGACGTTGAATGAAGGAGGA-3′ and 5′-TCACAGGCCTAGAACGATTG-3′) and sequence-specific TaqMan probes (*wsv459*, 5′-FAM-AGCACTGGCCGGCACGATC-3′-TAMRA; *wsv322*, 5′-FAM-C GTCTGGCTTCAGCGATTTATTGTCC-3′-TAMRA). Shrimp β-actin (primers, 5′-AGCAGATGTGGATCAGCAAG-3′ and 5′-GAAGCACTT CCTGTGAACGA-3′; TaqMan probe, 5′-FAM-TGATGGTCCAGACTCGTCATACTCCTG-3′-TAMRA) was used as a control. qRT-PCR was carried out as follows, 95°C for 30 s, soon afterward 40 cycles at 95°C for 5 s, and 60°C for 20 s.

### RNA Interference in Shrimp *In Vivo*

According to the *wsv459* and *wsv322* sequences, the *wsv459*-siRNA (5′-CCAAGAUGGCUUCGCCAUU-3′) specifically targeting the *wsv459* gene and the *wsv322*-siRNA (5′-GCCUGUGAAGCUGAUCCAU-3′) targeting the *wsv322* gene were synthesized using T7 Kit siRNA Synthesis (TaKaRa, Japan) *in vitro*. As a control, one randomly scrambled siRNA sequence siRNA-scrambled (5′-CCAAGAUGGCUUCACUCUU-3′) was used. The size of siRNAs was monitored by agarose gel electrophoresis. The synthesized siRNAs were dissolved in Tris buffer (50 mM Tris·HCl (pH 7.5), 100 mM NaCl), then quantified by NanoDrop 2000.

RNAi assay was carried out by injecting siRNA (30 μg/shrimp) into shrimp by syringes. First, siRNA (15 μg/shrimp) was injected into virus-free shrimp. Twelve hours later, WSSV (10^5^ copies/shrimp) and siRNA (15 µg) were co-injected into the same shrimp. At different time after the last injection, the shrimp hemocytes were harvested. Three samples of shrimp from each treatment, randomly selected, were harvested for experiments. The aforementioned assays were repeated three times for biological repetition.

### miRNA-Mediated Degradation of Target mRNAs

The shrimp *Ago1* gene was expressed in *E. coli*. Subsequently, the purified recombinant Ago1 protein was used to immunize mice to get the Ago1-specific antibody.

Cold RIPA (radio immunoprecipitation assay) lysis buffer (Beyotime Biotechnology, Shanghai, China) was added into shrimp hemocytes. After lysis at 4°C for 15 min, hemocytes were centrifuged at 14,000 × *g* (4°C) for about 15 min. The supernatant was collected. Then, Ago1-specific antibody and the protein G-coupled agarose beads (GE Healthcare, USA) were added into the supernatant, followed by incubation at 4°C overnight. The beads were rinsed with cold PBS for three times. To investigate the miRNA-mediated degradation of target mRNAs, the co-immunoprecipitated product of shrimp Ago1, WSSV-miRN-32, *wsv459* 3′UTR, or/and *wsv322* 3′UTR were mixed and incubated for 2 h at room temperature. The 3′UTRs of *wsv359* mRNA and *wsv322* mRNA were cloned with *wsv359*-specific primers (5′-GATCACTAATACGACTCACTATAGGGAGTCAGCGCATGC-3′ and 5′-AGGCTCGTACGCTGCGCGAG-3′) and *wsv322*-specific primers (5′-GATCAC TAATACGACTCACTATAGGGGGAGCTACGGAA-3′ and 5′-GATGCATCGAT CGTAGCCGCGCAT-3′), respectively. Then the 3′UTR of *wsv459* or *wsv322* was synthesized using a T7 Kit (TaKaRa, Japan). The mixture of Ago1 complex, WSSV-miRN-32, *wsv459* 3′UTR, or/and *wsv322* 3′UTR was electrophoresed on a 1% agarose gel after incubation. And then the RNAs were transferred to a Hybond-N+ (RPN303B) nylon membrane (GE Amersham, USA). After UV cross-linking, it was incubated in DIG Easy Hyb granule buffer (Roche, Switzerland) for 0.5 h at 42°C for prehybridization and then incubated in the same buffer with DIG-labeled *wsv459* probe (5′-AGCGATGCGCGCTAGACTAGTCGATCG-3′) or/and *wsv322* probe (5′-GCATCGCATGCTAGCGCGCATGCATC-3′) at 42°C overnight for hybridization. The alkaline phosphatase reaction was carried out by DIG DNA Labeling and Detection Kit II (Roche, Switzerland).

### Co-Localization of miRNA and mRNA

For co-localization analysis of viral miRNA and its target mRNAs, shrimp were infected with WSSV, and shrimp hemocytes were collected at 6 and 24 h postinfection. After rinses with PBS, the shrimp hemocyte cells were added onto poly lysine coated slides (Sigma-Aldrich, MO, USA) for adsorption for 30 min, followed by incubation with 4% paraformaldehyde for 30 min to fix hemocytes. The membranes of hemocytes were destroyed by incubation of hemocytes with 0.2% TritonX-100 for 30 min. Subsequently, DAPI (Sigma-Aldrich, MO, USA) and labeled probes (FAM-labeled WSSV-miR-N32 probe, 5′-FAM-AACGGACGTCACCTTC-3′; Cy3-labeled *wsv459* probe, 5′-Cy3-CAGAAGTGCAGTTGC-3′; Cy5-labeled *wsv322* probe, 5′-Cy5-TGGCTACCAGAACC-3′) were incubated on the slides. Two hours later, the fluorescence was quenched by trypan blue for 20 min, followed by PBS washes. Then, the hemocytes were visualized with a Zeiss 710 LSM microscope using HeNe laser excitation at 352 nm (DAPI), 488 nm (FAM), 543 nm (Cy3), and 633 nm (Cy5).

### Sequencing of the Cleaved mRNA 3′UTR Fragment

The mRNA 3′UTR of WSSV-miR-N32 target gene was incubated with WSSV-miR-N32 (1,000 nM) and Ago1 complex for different time (0.5, 1, or 2 h). After separation by agarose gel electrophoresis, the cleaved RNA fragment was recovered. Then, the reverse transcription was conducted using PrimeScript^®^ first strand cDNA synthesis kit with the Random 6 primer (TaKaRa, Shiga, Japan). The double-stranded DNA was synthesized by second strand cDNA synthesis kit (Beyotime Biotechnology, Shanghai, China). Subsequently, the dsDNA was ligated into T vector (TransGen Biotech, Beijing, China) and sequenced.

### Identification of Bases of a miRNA Non-Seed Sequence Required for miRNA Targeting

To identify the bases of a miRNA non-seed sequence required for miRNA targeting, 13th, 16th, 17th, 18th, 20th, 21st, or 22nd base of WSSV-miRN-32 (5′-GGGGAAGGUGACGUCCGUUUGA-3′) was mutated. The mutated WSSV-miRN-32 was synthesized using a T7 Kit siRNA Synthesis (TaKaRa, Japan) *in vitro*. The size of mutated miRNAs was detected by agarose gel electrophoresis. The synthesized miRNA was dissolved in Tris buffer (50 mM Tris·HCl (pH 7.5), 100 mM NaCl), then quantified by NanoDrop 2000. Virus-free shrimp were co-injected with WSSV (10^5^ copies/mL) and the wild-type or mutated WSSV-miRN-32 (15 µg) (100 µL per shrimp). 12 h after the first injection, another dose of miRNA (15 µg) was injected into the same shrimp. WSSV only group was used as a control group. 36 h after the last treatment, three samples of shrimp from every group, randomly selected, were harvested for experiments. The above experiments were repeated three times for biological repetition.

### Statistical Analysis

All numeric data gathered from above independent treatments were managed by one-way analysis of variation. Student’s *t*-test was used to check the significant differences between different treatments.

## Author Contributions

XZ designed the study and reviewed and edited the manuscript. XZ and YH performed the experiments and wrote the paper. All the authors read and approved the manuscript.

## Conflict of Interest Statement

The authors declare that the research was conducted in the absence of any commercial or financial relationships that could be construed as a potential conflict of interest.

## Footnotes

^1^http://pictar.mdc-berlin.de/.

^2^http://www.microrna.org/.

^3^http://www.targetscan.org.
